# Copper (II) binding of NAD(P)H- flavin oxidoreductase (NfoR) enhances its Cr (VI)-reducing ability

**DOI:** 10.1038/s41598-017-15588-y

**Published:** 2017-11-13

**Authors:** Huawen Han, Zhenmin Ling, Tuoyu Zhou, Rong Xu, Yongxing He, Pu Liu, Xiangkai Li

**Affiliations:** 1 Ministry of Education, Key Laboratory of Cell Activities and Stress Adaptations, School of Life Science, Lanzhou University, Tianshui South Road #222, Lanzhou, Gansu 730000 People’s Republic of China; 2 Department of Development Biology Sciences, School of Life Science, Lanzhou University, Tianshui South Road #222, Lanzhou, Gansu 730000 People’s Republic of China

## Abstract

Microbes can reduce hexavalent chromium Cr (VI) to the less toxic and soluble trivalent Cr (III). Copper stimulates microbial reduction of Cr (VI) by the *Bacillus*, *Ochrobactrum*, and *Gluconobacter* species; however, the mechanism remains unclear. In our study, the rate of Cr (VI) reduction by *Staphylococcus aureus* LZ-01 was increased by 210 % when supplemented with 60 μM Cu (II). A putative NAD(P)H-flavin oxidoreductase gene (*nfoR*) was upregulated under Cr (VI) stress. *NfoR-*knockout mutant displayed impaired reduction of Cr (VI) and Cu (II)-enhanced Cr (VI) reduction by *nfoR* isogenic mutant was attenuated in the presence of Cu (II). *In vitro* tests showed an increased *V*_*max*_ value of 25.22 μM min^−1^ mg^−1^ NfoR in the presence of Cu (II). Together, these results indicate that NfoR is responsible for Cu (II) enhancement. Isothermal titration calorimetry (ITC) assays confirmed the interaction of NfoR with Cu (II) at the dissociation constant of 85.5 μM. Site-directed mutagenesis indicates that His100, His128, and Met165 residues may be important for Cu (II) binding, while Cys163 is necessary for the FMN binding of NfoR. These findings show that Cu (II)-enhanced NfoR belongs to a new branch of Cr (VI) reductases and profoundly influences Cr (VI) reduction.

## Introduction

The characteristics of hexavalent chromium Cr (VI) include high infusibility, carcinogenicity, and broad-spectrum mutagenicity^1,2^. Cr (VI) bioremediation by microorganisms has recently attracted attention because it is more eco-friendly than other physicochemical treatments^3^. A growing body of empirical work suggests that many bacteria, such as *Bacillus* spp., *E*. *coli*, *Pseudomonas* spp., *Shewanella* spp., and *Staphylococcus* spp., can convert soluble and toxic Cr (VI) into insoluble and relatively non-toxic Cr (III)^4–7^. Several studies have attempted to optimize parameters, such as the initial Cr (VI) concentration, temperature, pH, and absorbance, to improve microbial Cr (VI)-reducing ability^8,9^. Recent studies show that divalent metal ions stimulate microbial Cr (VI) reduction and are important for the bioremediation of chromate pollution^10–12^. The Cr (VI)-reducing ability of the *Ochrobactrum sp*. strain CSCr-3 is significantly improved in the presence of Cu (II)^12^. However, while these studies show the importance of Cu (II), the exact mechanism of Cu (II)-enhanced Cr (VI) reduction remains elusive.

Chromate reductase is categorized as a member of membrane-associated and soluble chromate reductases (ChrR, YieF, NemA), which use NAD(P)H, or redox intermediates, such as FADH_2_, cysteine, ascorbate, and glutathione, as electron donors^13–17^. Several chromate reductases also act as iron reductases, nitroreductases, and glutathione reductases. Representative examples are the reductases NfsA from *Vibrio harveyi*, which acts as a nitroreductase and chromate reductase^18^. Most Cr (VI) reductases manifest flavoprotein activities, which bacteria use to catabolize numerous compounds^7,19,20^. Flavoproteins participate in various biological processes, such as dehydrogenation, oxidation, mono-oxygenation, and biological sensing processes^21^. The flavin cofactor, FMN or FAD, is non-covalently bound in the active site involved in the electron flow from the substrate to the acceptor^22^. For example, a crystal of *E*. *coli* ChrR is a tetramer, and the FMN cofactor plays a crucial role in the chromate-reducing activity of ChrR^23^. The activity of chromate reductase from *Thermus scotoductus* SA-01 has been found to be dependent on the divalent metal ions Ca (II) and Mg (II)^16^. Although previous studies show that Cu (II) is essential for Cr (VI) reductase activity in cell-free extracts of *Penicillium* spp., *Leucobacter* spp., and the *Bacillus* spp.^24–27^, this study examines the role of Cu (II) in microbial Cr (VI) reduction in order to understand its catalytic mechanism. We hypothesize that Cu (II) may specifically catalyze certain enzymes in cell-free extracts or exist as a novel Cu (II)-dependent chromate reductase involved in Cr (VI) reduction.

Cu (II) is a trace element essential for life in most prokaryotes; it performs multiple functions, particularly that of a cofactor to numerous proteins^28^. Cu (II) exhibits a preferred coordination to oxygen or imidazole nitrogen groups from aspartic and glutamic acid, or histidine, respectively, while metallothioneins have a higher affinity for Cu (I)^29^. Typically, Cu (II)-dependent enzymes, including lactase, nitrite reductase, and monooxygenase, reside in the cytoplasmic membrane or periplasm, where they are loaded with Cu (II)^30–33^. For example, a copper-dependent polysaccharide monooxygenase (GH61), bound to Cu (II), enhances the activity of cellobiose dehydrogenase (CDH) and accelerates the oxidation of cellobiose^34^. Cu (II) and Fe (III) substantially enhance the activity of chromate reductase purified from *Ochrobactrum sp*. Cr-B4^35^. Similar phenomena are observed in a number of strains within the *Bacillus* genus^36^. However, there are no reports on the chromate-reducing catalytic mechanism of Cu (II). Thus, examining potential coordination between Cu (II) and the purified enzyme may lead to enhancement in the activity of the enzyme.

*Staphylococcus aureus* LZ-01 is isolated from the sediments of Yellow River in the Lanzhou area^37^. We show that Cu (II) markedly improves the Cr (VI)-reducing ability and Cr (VI) resistance of *S*. *aureus* LZ-01. This synergy is caused by the capacity of Cu (II) to stimulate the enzymatic activity of the strain LZ-01. Using transcriptome analysis, we screened for the chromate reduction-related gene, *nfoR*, which encodes a putative NAD(P)H-flavin oxidoreductase (NfoR), in strain LZ-01. Isogenic mutant analysis showed that *nfoR* differs from the known Cr (VI) reductases. *In vitro* analysis showed that *nfoR* is responsible for Cu (II)-induced enhancement, and that the residues H100, H128, and M165 are the potential sites for Cu (II) binding. In this study, we examined the different electron transfer processes of NfoR. These results may expand our knowledge of Cu (II)-induced enhancement and benefit the Cr (VI) bioremediation.

## Results

### Cu (II) promotes the Cr (VI)-reducing ability of *S*. *aureu*s LZ-01

**Figure 1:**
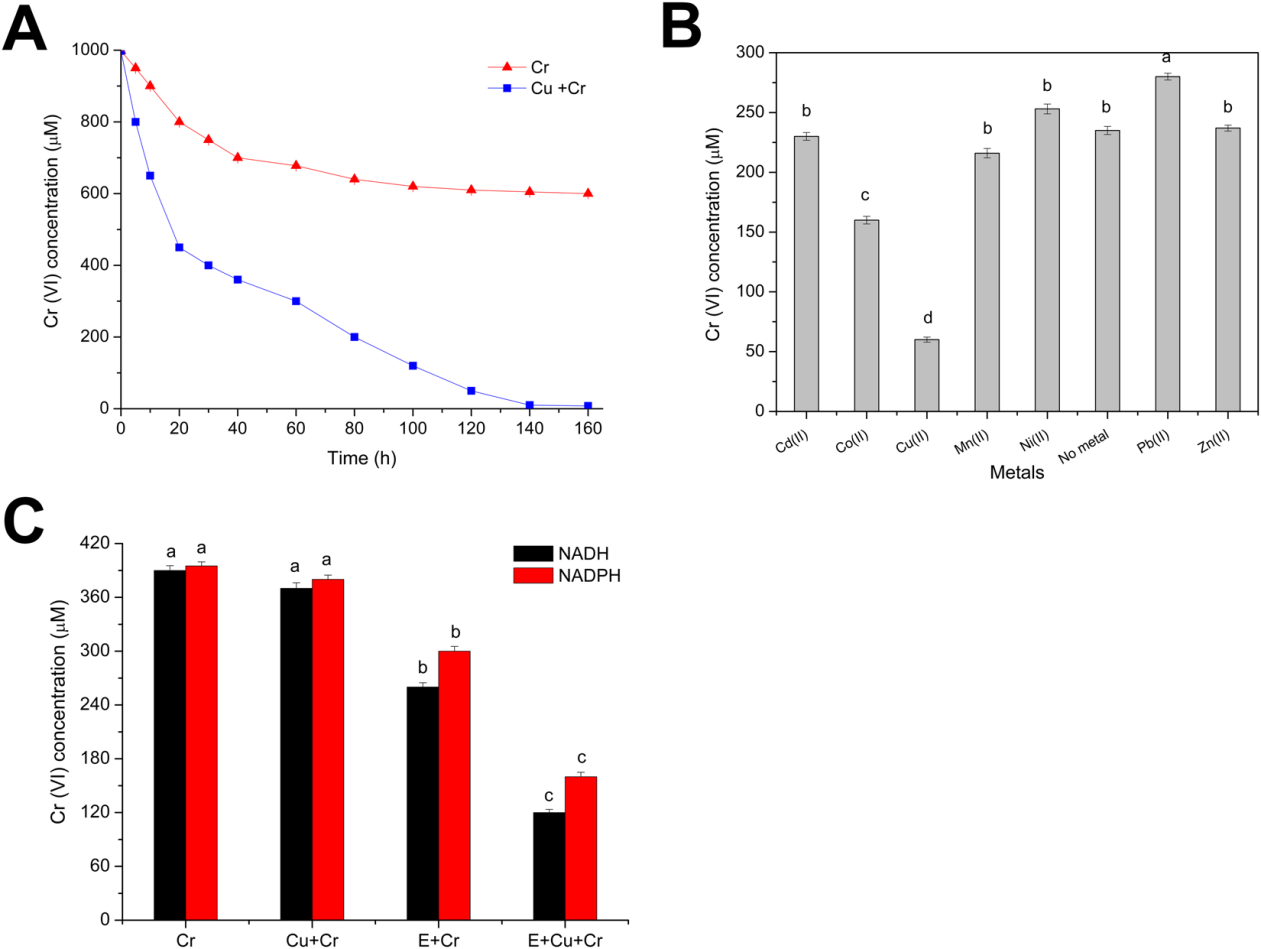
(**A**) The Cr (VI) reduction curve of *Staphylococcus aureus* LZ-01, incubated with 1 mM Cr (VI), and supplemented with 0 μM Cu (II) and 60 μM Cu (II), in M9 medium. (**B**) The effects of different divalent heavy metals ions on Cr (VI) reduction by LZ-01 resting cells (an OD_600_ of 1.0) incubated in 0.85% NaCl containing 400 µM Cr (VI), 0.3 mM NADH, supplemented with 0 μM Cu (II) or 60 μM Cu (II), and incubated for 24 h. (**C**). Chromate reductase activity in cell-free extracts (**E**) of LZ-01 in the presence of Cu (II). The reaction contained 20 mM Tris-HCl buffer (pH 7.0), 50 µM E, 0.3 mM NAD(P)H, or 60 µM Cu (II) at the initial concentration of 400 μm Cr (VI). (**E**) The crude enzyme of *S*. *aureus* LZ-01 was induced overnight by 200 μM Cr (VI). Error bars indicate three replicates.

To investigate the role of Cu (II) in Cr (VI) reduction by *S*. *aureu*s LZ-01, we compared the Cr (VI) reduction curve alone and combined with Cu (II) (Fig. 1A). Cu (II) significantly promoted Cr (VI) reduction and increased the reduction rate under the growth conditions, indicating that Cu (II) plays a prominent role in Cr (VI) detoxification. Treatment with Cu (II) showed a similar result in the resting cell experiments (Fig. 1B). Moreover, a slight improvement in Cr (VI)-reducing activity occurred in the presence of Co (II), while other metals did not affect the bacterial reduction of Cr (VI). The addition of Cu (II) affected the enzymatic activity of Cr (VI) reduction in cell-free extracts (Fig. 1C), suggesting that Cu (II) directly or indirectly acts on the enzymes or regulatory elements involved in Cr (VI) reduction.

### *nfoR* is responsible for Cu (II)-enhanced Cr (VI) reduction

**Figure 2:**
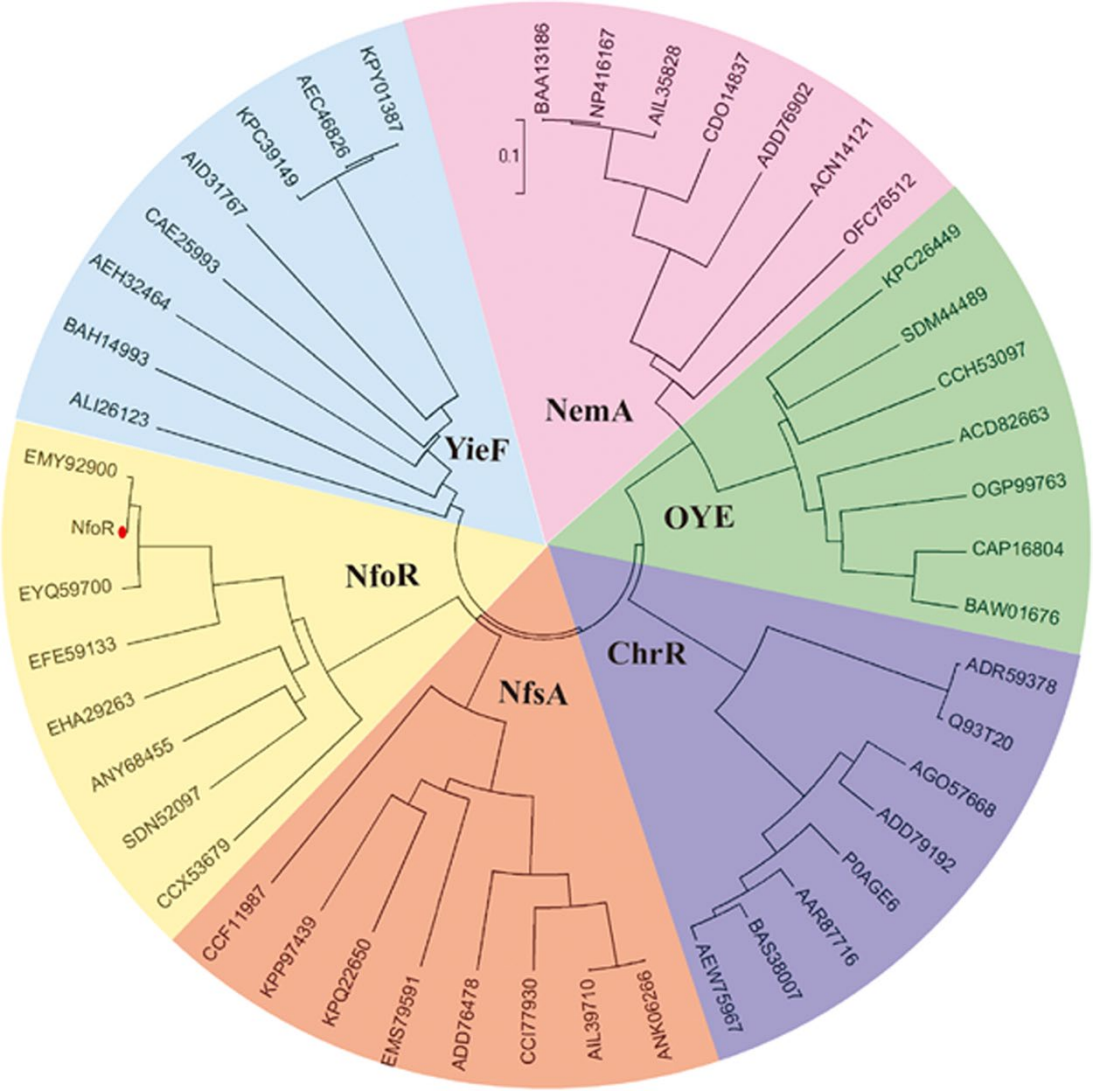
Evolutionary relationship of NfoR with known chromate reductases from different genera. Composite evolutionary tree with the NfoR is shown as a Newick-like cladogram for clarity and includes other chromate reductase families (ChrR, YieF, NfsA, OYE, and NemA).

To ascertain the potential involvement of chromate reductase in Cr (VI) reduction by LZ-01, we conducted a comparison analysis of 512 genes upregulated under Cr (VI) stress, based on our previous transcriptome analysis^7^. According to previous studies, we speculated five candidate genes (*nfoR*, *nfsA*, *trxB*, *nemA*, and *ndh*) may be involved in Cr (VI) reduction in the strain LZ-01 based on amino acid sequence alignment^13,18,38,39^. Figure S1 presented these gene expression under Cr(VI) stress. A phylogenetic comparison of the protein products of these five genes was conducted using the NJ method with different genera. The predicted NfoR (a putative NAD(P)H-flavin oxidoreductase) was shown to belong to a distinct clade, separate from the known chromate reductases (Fig. 2).

**Figure 3:**
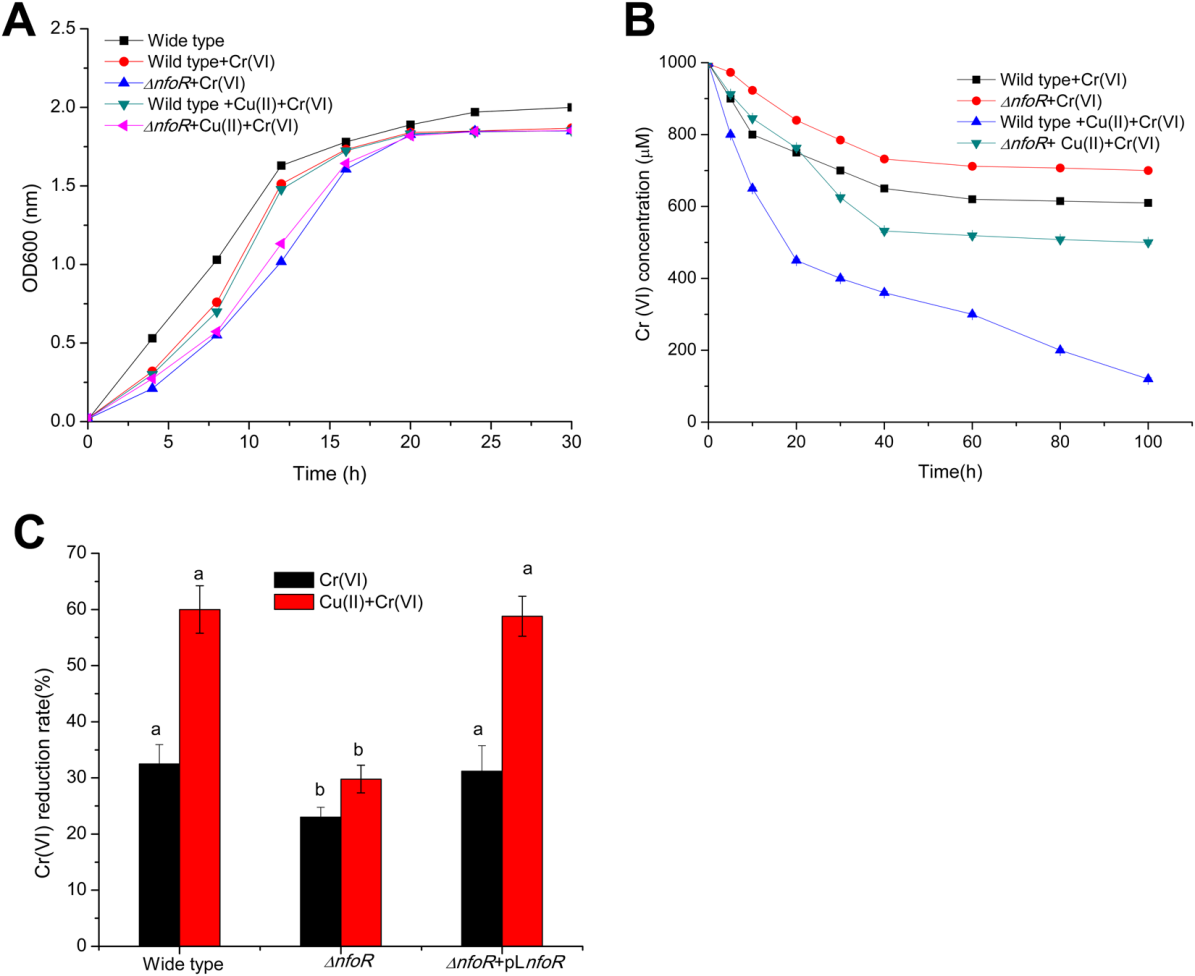
(**A**) Growth curve of wild type (WT) *S*. *aureus* LZ-01 and Δ*nfoR* mutant, grown aerobically in M9 medium in the absence or presence of 1 mM Cr (VI) or 60 μM Cu (II). (**B**) Cr (VI) reduction rate of WT *S*. *aureus* LZ-01 and Δ*nfoR* mutant, grown aerobically in M9 medium with 1 mM Cr (VI) or 60 μM Cu (II). (**C**). Cr (VI) reduction rate of Δ*nfoR* mutant, and its gene complementation strain Δ*nfoR* + pL*nfoR*, grown aerobically in M9 medium with 1 mM Cr (VI) or 60 μM Cu (II) for 30 h.

To examine the functions of the five genes *in vivo*, we used an *nfoR* knockout mutant, which displays a slightly delayed growth under Cr (VI) stress and an impaired Cr (VI)-reducing ability (Fig. 3A). The *nfoR* knockout mutant can’t completely lose its reducing ability because of other chromate reductases present in the strain LZ-01 (Fig. S1). Cu (II)-enhanced Cr (VI)-reducing ability from *nfoR* isogenic mutant was attenuated in the presence of Cu(II) (Fig. 3B). Although we found these three mutants (*nfsA*, *trxB* and *nemA*) decreased the Cr (VI) reduction ability, they were not responsible for Cu (II) enhancement (data not shown). In fact, our previous data demonstrated the NfsA, TrxB and NemA are involved in the Cr (VI) reduction, which is in accordance with the results of the previous studies. However, their enzyme activity can’t be stimulated by Cu (II) (Fig. S2). In addition, NDH belongs to a membrane-associated protein with Cr(VI)-reducing activity, we failed to purify this enzyme^39^. Gene complementation strain *ΔnfoR* + pLnfoR have similar Cr (VI) reduction rate with wild type in the absence or presence of Cu (II), and their Cr(VI) reduction ability can both be enhanced by Cu (II) (Fig. 3C). Thus, NfoR is essential for Cr (VI) resistance and reduction by *S*. *aureus* LZ-01 under Cr (VI) stress, and contributes to the enhanced Cr (VI) reduction stimulated by Cu (II).

### Enzyme kinetics of NfoR in Cr (VI) reduction

NfoR was overexpressed in *E*. *coli* Rosetta (DE3) cells with typical yields of 80 mg of purified protein/liter of bacterial culture. The molecular weight of the NfoR, as detected by SDS-PAGE, was approximately 26.4 kDa with FMN as a cofactor (Figs S3 and S4). Subsequently, to quantitatively detect the percent of the isolated NfoR that retains the bound FMN, the correlations between the FMN concentrations and the mAu showed a linear relationship (*R* ^2^ = 0.9958). According to the mAu value from NfoR supernatant, the percent of FMN/isolated NfoR was 79.98% (Fig. S5). The tightness of FMN span a wide range in the family of NAD(P)H-flavin oxidoreductases^40^. To solve this issue, supplementation free FMN has no significant effect on NfoR activity, indicating that NfoR combined with the saturated FMN during isolation (Fig. S6).

**Figure 4:**
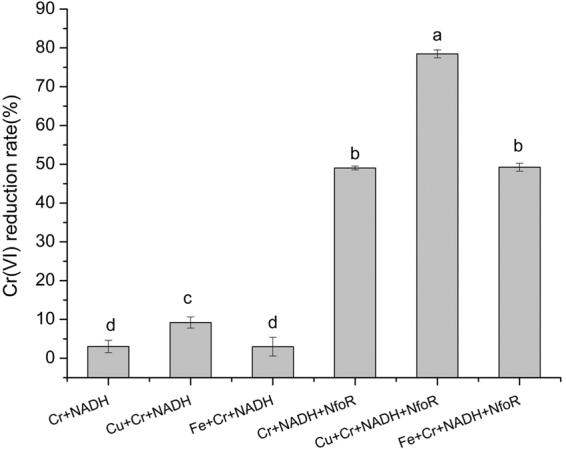
Cu (II) enhanced the chromate reduction rate of purified NfoR. Reaction mixtures (1 mL) containing 20 mM Tris-HCl buffer (pH 7.0), 20 μM NfoR, 0.3 mM NADH, 0.2 mM Cr(VI), and 50 μM Cu(II) or Fe (III), were incubated for 10 min in 37 °C. Error bars indicate standard errors.

The optimal pH and temperature for NfoR were 7.0 and 37 °C, respectively. NfoR has same preference for NADH and NADPH as electron donor (data not shown). However, some references reported that NemA specificity for NADH over NADPH^13^. Moreover, Cu (II) can significantly improve the Cr (VI)-reducing rate of NfoR (Fig. 4). To eliminate the possibility that Cu (II) catalyzed the chemical reaction between Cr (VI) and NADH, we established that the rate of Cr (VI) reduction, after treatment with Cu + Cr + NADH + NfoR, was superior to that of the control (Cu + Cr + NADH); this indicates that Cu (II) can accelerate the electron transfer from NADH to Cr (VI). Although Hora described supplementation of Fe (III) substantially enhanced the activity of chromate reductase purified from *Ochrobactrum sp*. Cr-B4^35^, Fe (III) has no significant effect on NfoR activity (Fig. 4).

**Figure 5:**
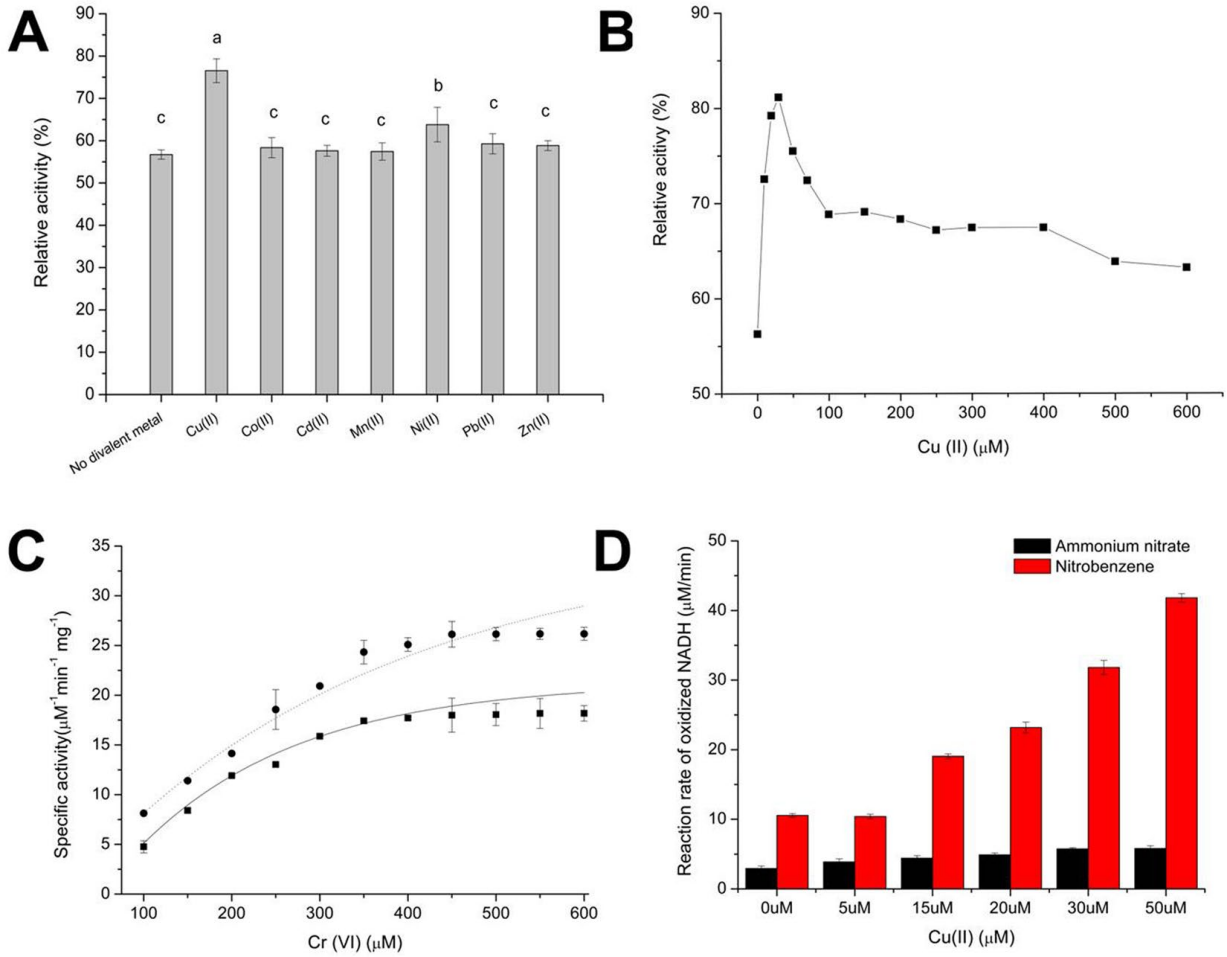
(**A**) The effects of different divalent metals on Cr (VI)-reducing ability of NfoR. (**B**) NfoR stability under different concentrations of Cu (II). Reaction mixtures (1 mL), containing 20 mM Tris-HCl buffer (pH 7.0), 25 μM enzyme, 0.3 mM NADH, 0.2 mM Cr (VI), and various concentrations of Cu (II), were incubated for 3 min at 37 °C. (**C**) Steady-state kinetics of NfoR for Cr (VI) reduction supplemented with 0 μM Cu (II) (▪) or 60 μM Cu (II) (⦁). (**D**) Assessment of the nitroreductase activity of NfoR using nitrobenzene and ammonium nitrate as electron acceptors. Reaction mixtures (500 μl), containing 50 mM Tris-HCl buffer (pH 7.0), 10 μM NfoR, 0.2 mM NADH, 100 μM ammonium nitrate or nitrobenzene, and different concentrations of Cu (II), were incubated for 8 min at 28 °C. Error bars indicate standard errors.

We examined whether other divalent metals similarly enhance the activity of NfoR. NfoR activity increased 1.5 fold in the presence of Cu (II), whereas only a slight improvement was observed after treatment with Ni (Fig. 5A). NfoR showed increased activity with an increase in Cu (II) concentration; its Cr (VI)-reducing ability reached a maximum (82%) when 30 µM Cu (II) was added to the reaction mixture (Fig. 5B). The *K*_*m*_ and *V*_*max*_ values of NfoR for Cr (VI) reduction were 120.84 μM and 16.30 μM·min^−1^·mg^−1^ under untreated conditions while the *K*_*m*_ and *V*_*max*_ values of NfoR for Cr (VI) reduction were 150.50 μM and 25.22 μM min^−1^ mg^−1^ in the presence of 30 μM Cu (II), respectively (Fig. 5C). NfoR could utilize ammonium nitrate and nitrobenzene as substrates, with a preference toward nitrobenzene (Fig. 5D). Similarly, Cu (II) enhanced the ammonium nitrate and nitrobenzene reduction by NfoR, with maximum reaction rates of 41.8 ± 0.62 mM min^−1^ and 5.82 ± 0.38 mM min^−1^, respectively. These results show that Cu (II) specifically catalyzes and enhances the activity of NfoR.

### Cu (II) changes the conformation of NfoR

**Figure 6:**
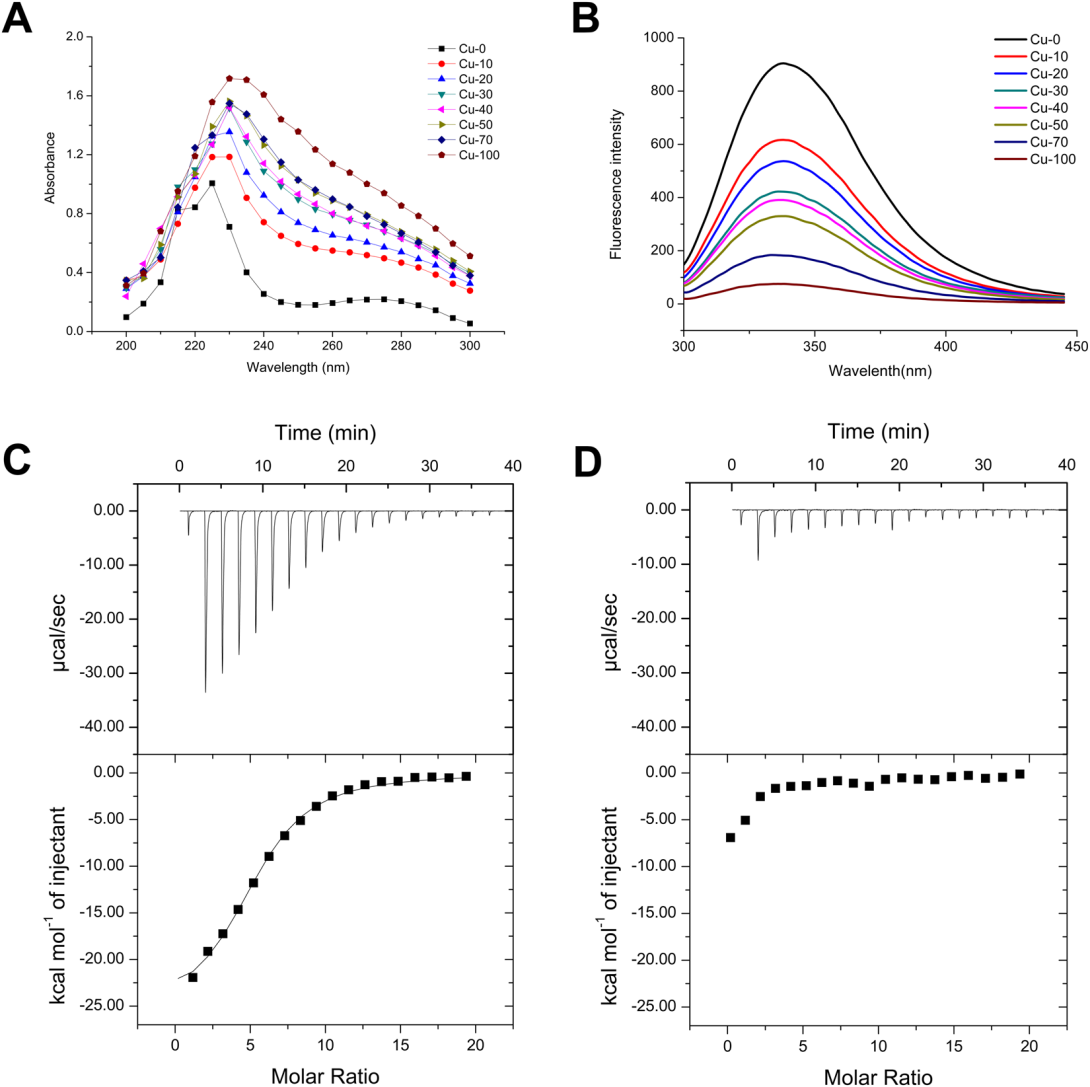
(**A**) UV-Vis absorbance spectra of NfoR in the presence of Cu (II). Reaction mixtures (1 mL), containing 20 mM Tris-HCl buffer (pH 7.0), 5 μM NfoR, and different concentrations of Cu (II), were incubated for 30 min at 37 °C. (**B**) Fluorescence emission spectra of NfoR, excited at 280 nm in the presence of Cu (II), NfoR concentration (5 × 10^−6^ mol L^−1^), Cu (II) concentration (0, 10, 20, 30, 40, 50, 70, or 100 × 10^−6^ mol L^–1^). (**C**,**D**) Isothermal calorimetry (ITC) analyses of NfoR. Approximately 10 mM Cu (II) (**C**) and Ni (II) (**D**) were injected into 0.1 mM NfoR, respectively.

The acceleration of NfoR activity by Cu (II) can be potentially ascribed to the interaction between NfoR and Cu (II). To validate this hypothesis, spectral experiments were conducted to capture the structural changes induced by the various Cu (II) concentrations. UV absorption intensity, reflecting changes in NfoR with respect to Cu (II) concentrations, is shown in Fig. 6. A red shift of the maximum peak of NfoR occurred at 225 nm because of the formation of a complex between NfoR and Cu (II) (Fig. 6A). Supplementation with various concentrations of Cu (II) significantly decreased the fluorescence intensity of NfoR, indicating the binding of NfoR to Cu (II) (Fig. 6B). The peak of NfoR shifted from 280 to 285 nm after treatment with Cu (II), suggesting that the microenvironment around the Tyr residues had discernibly changed during the binding process (Fig. S7). Conversely, Ni (II) showed no obvious impact on the fluorescence intensity of NfoR, indicating Cu (II) specifically changed the conformation of NfoR (Fig. S8). We then used ITC to further characterize the interaction of NfoR with Cu (II) and Ni (II) (Fig. 6C, 6D). NfoR bound Cu (II) at a stoichiometric ratio of 1:5.26 and a dissociation constant of 85.5 μM. Subsequently, fluorescence spectra experiments combined with ITC analysis further demonstrated that NfoR can bind Fe (III) (Fig. S9), but there show no stimulation on NfoR activity, indicating that NfoR exists a non-specific Fe (III) binding site with a distinction from Cu(II) binding site. In addition, consistent with the spectral data, NfoR did not detect with Ni (II) binding (Table S3). These results demonstrate that Cu (II) can be a ligand for NfoR and change the conformation of NfoR.

### Residues of NfoR that potentially bind Cu (II)

**Figure 7:**
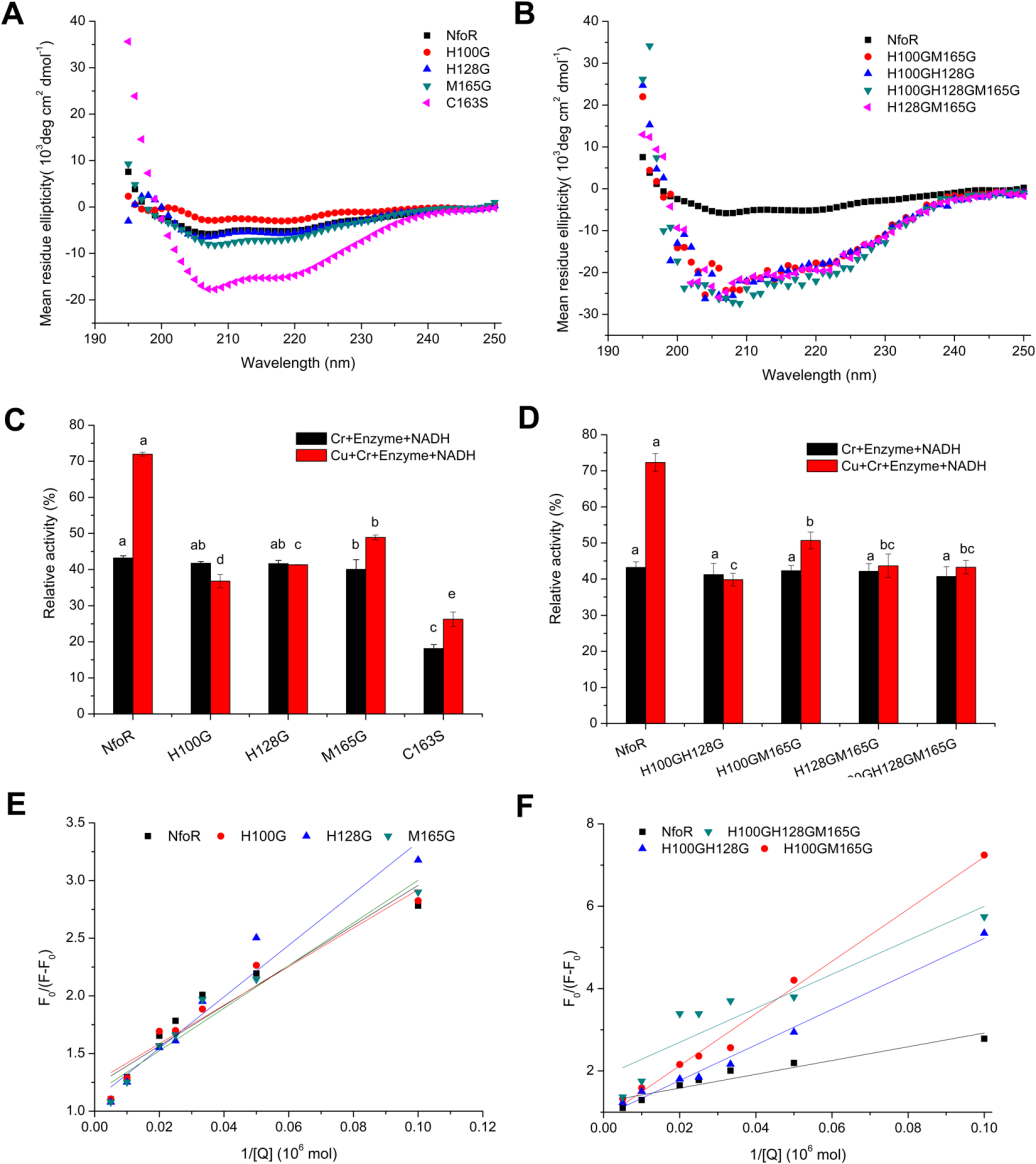
(**A**,**B**) Circular dichroism spectra of native NfoR and its mutants in the far UV region. (**C**,**D**) Relative activity of purified NfoR and its mutants. Reaction mixtures (1 mL), containing 50 mM Tris-HCl buffer (pH 7.0), 25 μM enzyme, 0.3 mM NADH, 0.2 mM Cr (VI), and 30 μM Cu (II), were incubated for 5 min at 37 °C. Error bars indicate standard errors. (**E**,**F**) The Stern-Volmer curve of the Cu (II)-NfoR and Cu (II)-NfoR mutants at 298 K, pH 7.0.

To better understand the potential binding residues, involved in the binding of Cu (II) with NfoR, it is essential to explore the models of Cu (II)-dependent proteins^41^. Based on the alignment of NfoR homologues, we found that the four residues His100, His128, Cys163, and Met165 were different from those present in the known chromate reductases (Fig. S11). Thus, H100, H128, and M165 were mutated glycine, while C163 was mutated serine. The purified NfoR mutants showed a yellow coloration, except for C163S, indicating that C163 is a key residue in the binding of FMN (Figs S4D and S12). We found that mutations in H100, H128, and M165 had no obvious effect on the activity of NfoR, but attenuated the Cu (II)-enhanced effect (Fig. 7C). C163S abolished the activity of NfoR, possibly because of the dissociation of the FMN ligand. However, this attenuation was partially restored in the presence of Cu (II) (Fig. 7C). CD spectra from 190 to 250 nm did not indicate significant differences in the secondary structures of single mutants, with the exception of C163S, while multiple mutations significantly changed the secondary structures (Fig. 7A, 7B). As shown in Fig. 7D, multiple mutations showed no additive effect on the activity of NfoR in spite of the conformational change (Fig. 7B). The Stern-Volmer curve, generated to evaluate the key residues of NfoR involved in Cu (II) binding, showed that point mutations decreased the *K*_*a*_ values between NfoR and Cu (II) (Fig. 7E, 7F). Other divalent metals had no obvious impact on the relative activity of NfoR mutants, indicating that these residues are involved in Cu (II) binding (Fig. S13). Hence, H100, H128, and M165 are the potential binding residues involved in Cu (II) enhancement of the activity of NfoR.

## Discussion

Our finding that Cu (II) can improve the Cr (VI)-reducing ability of *S*. *aureus* LZ-01 agrees with previous studies describing the role of the Cu (II) enhancement during Cr (VI) bioremediation. These studies have shown that the presence of Cu (II) and Co (II) significantly enhances the Cr (VI)-reducing ability of *O*. *intermedium*, while Zn (II) and Pb (II) showed the strongest inhibitory effect^10^. Similarly, Cu (II) increased the rate of Cr (VI) reduction by *Brevibacterium* sp. K1 and *Stenotrophomonas* sp. D6 to 160% and 192%, respectively^42^, and that of LZ-01 to 210%. The activity of chromate reductase in cell-free extracts of LZ-01 was also substantially stimulated by Cu (II) (Fig. 1C). Cu (II) may function as a prosthetic factor for numerous reductases or as an electron transporter between protein subunits^43^. This result is supported by a study by Soni *et al*., showing that enzymatic activities in the cell-free extracts of the *Bacillus* spp. are improved in the presence of Cu (II), with specific activity reaching 0.44 ± 0.098 μM min^−1^ mg^−1^ ^44^. Although Cu (II)-enhanced reduction of Cr (VI) is common in environmental microbes, no enhancement in the activity of crude chromate reductase in the *Arthrobacter* sp. SUK 1201 was observed after the addition of Cu (II)^45^.

**Figure 8:**
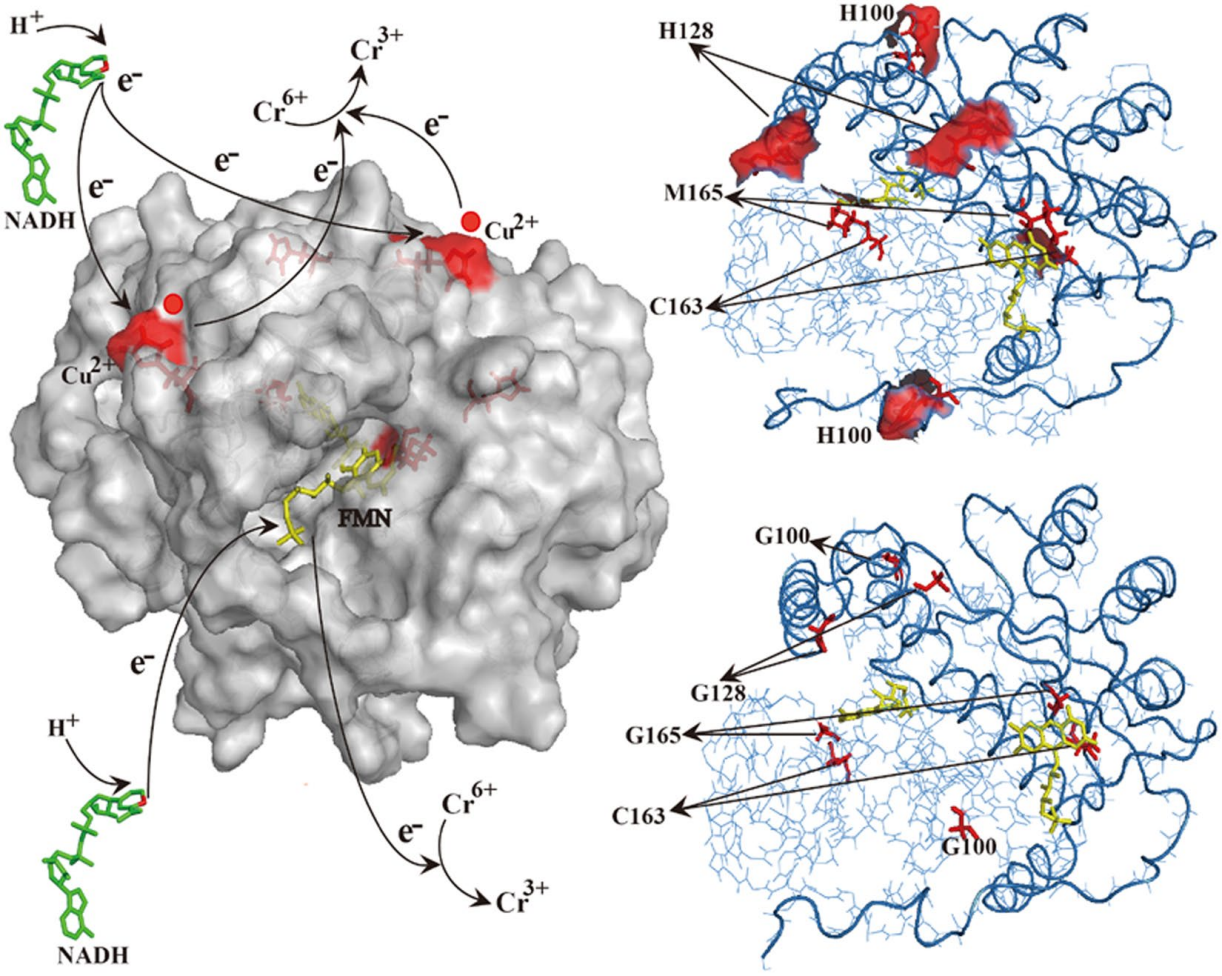
Cu (II)-enhanced bacterial reduction of Cr (VI). This diagram depicts a proposed mechanistic model for Cu (II)-mediated enhancement of NfoR activity in the reduction of Cr (VI). Cu (II) can bind the specific site of NfoR to accelerate the reduction of Cr (VI). Cu (II), as an electron intermediate, replaces the function of the FMN cofactor in the NfoR protein, resulting in enhanced bacterial survival and detoxification of Cr (VI).

Previous studies, using global transcriptome and proteomic analyses, have identified only the enzyme or protein types involved in Cr (VI) resistance and reduction mechanisms^7,46^. Using the transcriptional profile of the strain LZ-01, under Cr (VI) stress, we identified five candidate chromate reductases (NemA, NfsA, TrxB, Ndh, and NfoR)^13,47^. The *nfoR* knockout mutant is not susceptible to the stimulatory effect of Cu (II), which is consistent with the phylogenetic trees of different chromate reductase families (Fig. 2). This phenotype indicates that NfoR belongs to a novel branch of chromate reductases. Besides, supplementation of Cu (II) can slightly stimulate *ΔnfoR* mutant to reduce Cr (VI) compared with the treatment in the absence of Cu (II). Thus, the strain LZ-01 must exist one or more of the remaining reductases must be responding to Cu (II). In a follow-up study, we screen out another chromate reductase from LZ-01 involved in Cu (II) enhancement and the presence of Cu (II) can induce this gene expression (data not shown). Structural modeling of NfoR, using a putative NAD(P)H-flavin oxidoreductase as a crystal structure template (PDB code 2HAY), showed that it is also a dimer bound with a flavin mononucleotide (FMN) per subunit (Fig. 8). The values of *K*_*m*_ and *V*_*max*_ of NfoR during Cr (VI) reduction were 120.84 μM and 16.30 μM min^−1^mg^−1^, respectively (Fig. 5C). Two electrophoretic flavoproteins, ChrR and YieF, showed similar kinetic parameters during Cr (VI) reduction (*kcat/K*_*m*_ = 2 × 10^4^ M^−1^ s^−1^)^48^. Likewise, a novel chromate reductase, related to old yellow enzyme (OYE) involved in Cr (VI) reduction, shows corresponding *K*_*m*_ and *V*_*max*_ values of 3.5 μM and 6.2 μM min^−1^mg^−1^, respectively^16^. The finding that Cu (II) can accelerate the *V*_*max*_ of NfoR in response to Cr (VI) reduction agrees with the report describing the activity of the nitroreductase Gox0834 from *Gluconobacter oxydans* ^49^.

Interestingly, Cu (II) and Fe (III) appear to affect the allosteric conformation of NfoR (Fig. 5, Fig. S8). ITC analysis indicated that NfoR can directly bind Cu (II) and Fe (III), which agrees with similar results of a previous study^50^. The main difference is that the presence of Fe (III) has no stimulation on NfoR activity. In view of that NfoR can both bind with Fe(III) and Cu (II), coexistence of Fe (III) and Cu(II) have no obvious impact on NfoR activity compared to the treatment 50 μM Cu + Cr + NADH + NfoR, further suggesting there has no competition between Fe (III) and Cu(II) (Fig. S10). Thus, the prerequisite was defined to analyze potential Cu (II)-binding residues of NfoR. Previous studies confirmed that the terminal NH_2_ (Cu-N, 2.2 Å) and the *p*-N of the N-terminal histidine (Cu-N, 1.9 Å) of lytic polysaccharide monooxygenase can coordinate with Cu (II)^51^. Comparison of the active site residues of the Cu (II)-loaded protein structure showed a structural overlap with all the atoms of His, Met, Tyr, and Cys^41,51^. We found that mutations in H100, H128, and M165 suppressed Cu (II)-mediated activity of NfoR (Fig. 7B), which has similar result to those found in other characterized azurin^33^. The mutations in H100, H128 and M165 decreased the exposure of the residues, which is not conducive for Cu (II) binding (Fig. 8). Interestingly, Cu (II) can partially restore the activity of the C163S mutant because of the deficiency in the FMN cofactor (Fig. S3D). FMN is essential for the process of flavoprotein electron transfer from substrates to the terminal electron acceptors^52^. We showed that Cu (II) acts as an electron intermediate of chromate reductase, thereby resulting in enhanced activity of NfoR (Fig. 8). However, the mechanism, describing the transportation of electrons, needs to be further validated via cocrystallization of NfoR and Cu (II).

The evolutionary relationship of NfoR with other chromate reductase families (ChrR, YieF, NfsA, NemA, and OYE) showed that NfoR sequences are strongly separate from other chromate reductase groups, indicating that it belongs to a new clade of chromate reductases. Because NfoR groups are closely related, we hypothesize that the Cu (II)-enhanced effect is conserved in EMY92900 (*Staphylococcus*), EHA29263 (*Bacillus*), CCX53679 (*Veillonella*), and SDN52097 (*Fictibacillus*). These results, combined with our enzymatic studies of NfoR, suggest that NfoR exhibits two independent electron transfer channels in the process of Cu (II)-enhanced Cr (VI) reduction (Fig. 8). As an environmental factor, Cu (II) can directly trigger NfoR activity to improve Cr (VI) reduction and resistance, which is already present in microorganisms surviving in harsh environments. An evolutionary advantage of Cu (II)-enhanced Cr (VI) reduction may be that microorganism tolerance of Cr (VI) toxicity can be enhanced by Cu (II) via direct or indirect activation of Cr (VI) detoxification^53,54^. Considering the relative broad-spectrum substrates of NfoR, Cu (II)-mediated NfoR may protect against other organic pollutants such as nitrocompounds. Therefore, bacteria, such as LZ-01, can be used for Cr (VI) and organic waste removal in industrial wastewater containing various heavy metals. Furthermore, if this process exists in other plant growth-promoting rhizobacteria in the soil, it enhances the survival of beneficial bacteria and ameliorates contaminated soil.

## Materials and Methods

### Bacterial strains and growth conditions

The bacterial strains and plasmids, used in this study, are listed in the Supplementary Table S1. The oligonucleotides, used in the strain and plasmid construction, are listed in Supplementary Table S2. *Escherichia coli* DH5α and Rosetta (DE3) were cultivated in LB medium with 100 μg·mL^−1^ ampicillin, 50 μg·mL^−1^ kanamycin, or 30 μg·mL^−1^ chloramphenicol. *Staphylococcus aureus* LZ-01 and *S*. *aureus* RN4220 were grown in tryptic soy broth (TSB) medium with 5.0 μg·mL^−1^ erythromycin or 50 μg·mL^−1^ kanamycin, added as necessary.

### The role of Cu (II) in Cr (VI)-reducing ability of strain LZ-01

The strain LZ-01 (1 mL), cultured overnight, was inoculated into 100 mL of M9 minimal medium, supplemented with 1000 µM Cr (VI) or 60 µM Cu (II), and incubated for 7 days at 37 °C. M9 minimal salts medium was composed of 17 mM Na_2_HPO_4_ (AR), 22 mM KH_2_PO_4_ (AR), 19 mM NH_4_Cl (AR) and 0.1% yeast extract (AR) (w/v). The pH was adjusted to 7.4 prior to autoclaving and 2 mM sterilized MgSO_4_ (AR) and 0.2% (w/v) glucose (AR) were added after autoclaving^37^. The residual Cr (VI) concentration in the supernatant was measured as described previously^4^. Similarly, the resting cell experiment was used to investigate the effects of various divalent metals on the Cr (VI)-reducing ability of LZ-01. Briefly, the resting bacterial cells were harvested and adjusted to an OD_600_ of 1.0 in 20 mL 0.85% NaCl, supplemented with Cr (VI), or other divalent metals (Cu, Co, Ni, Cd, Mn, Zn, Pb), to the final concentrations of 400 µM and 60 µM, respectively, and using sodium lactate as an electron donor. After 24-h incubation, the concentration of residual Cr (VI) in the supernatant was measured. Cell-free extracts (E) from LZ-01 were prepared as described previously^26^. The enzyme activities in (E) were detected using 2-mL reaction mixtures containing 20 mM Tris-HCl buffer (pH 7.0), 50 µM E, 0.3 mM NADH, 400 µM Cr (VI), or 60 µM Cu (II). The reaction was started by adding Cr (VI) to the reaction mixture; the residual chromate concentration was measured after 30 min. Controls without E were also assayed. The concentration of total proteins was determined using a BCA kit (Takara) with bovine serum albumin used as standard.

### Construction of the Δ*nfoR* mutant

Construction of the Δ*nfoR* mutant was performed as described previously^55^. Briefly, the *nfoR* isogenic mutant was constructed, using homologous recombination, by ligating three fragments (upstream of the *nfoR*, kanamycin marker, downstream of *nfoR*) into the selective plasmid pMAD. The recombinant plasmid can be further modified in *S*. *aureus* RN4220 and transformed into *S*. *aureus* LZ-01. The strains, with allelic replacement of *nfoR* by *kan*, were selected as kanamycin-resistant and erythromycin-sensitive colonies. Strains with deleted *nfoR* were verified using colony PCR and sequencing. For the complementation of the Δ*nfoR* mutant, a 1024-bp fragment of the *nfoR* ORF, comprising the promoter region, was cloned into pLI50 to generate pLInfoR, which was electro-transformed into strain RN4220 and subsequently introduced into strainLZ-01Δ*nfoR*.

### Expression and purification of His-tagged NfoR

The *nfoR* gene was amplified and cloned into pET-28a, and the protein was expressed with N-terminal 6 × His tag. The recombinant plasmid, pEnfoR, was confirmed by DNA sequencing and transformed into the expression strain *E*. *coli* Rosetta (DE3).

The same purification procedure was used for both proteins. Bacterial cells were grown in 500 mL Terrific Broth (TB) medium, with 50 µg·mL^−1^ kanamycin and 30 µg·mL^−1^ chloramphenicol, at 37 °C. TB medium contained (in 1 liter) 12 g of tryptone (AR), 24 g of yeast extract (AR), 4 ml of glycerol, 2.31 g of KH_2_PO_4_, and 12.54 g of K_2_HPO_4_^56^. In the logarithmic phase, 0.5 mM isopropyl β-D-1-thiogalactopyranoside (IPTG) was added to the culture, and the culture was incubated at 16 °C for 20 h. The cells were harvested by centrifugation at 8000 *g* for 10 min and suspended in binding buffer (20 mM Tris-HCl pH 8.0, 100 mM NaCl, and 10 mM imidazole). His-tagged NfoR was purified with a HisTrap FF crude column (GE Healthcare Life Sciences) using different buffers (washing buffer: 20 mM Tris-HCl pH 8.0, 100 mM NaCl, and 20 mM imidazole; elution buffer: 20 mM Tris-HCl pH 8.0, 100 mM NaCl, and 300 mM imidazole). NfoR was analyzed by SDS-PAGE, and a Centrifuge Biomax-5 column (Millipore, Billerica, MA, USA) was used for desalting, followed by storage at −80 °C until use.

### Flavin composition of NfoR and NfoR ΔC163S

For flavin assays, concentrated protein samples were heated up to 95 °C for 10 min (water bath), and the released flavin was separated from the protein by centrifugation (12000 *g*, 10 min, 4 °C). The supernatant was then analyzed by HPLC (Agilent, Germany) using a ZORBAX SB-C18 column (16 mm × 150 mm) with an eluent of methanol and ammonium acetate (5 M/L, pH 6.0) at a flow rate of 1.0 mL/min. In order to quantitatively measure the percent of the isolated NfoR that retains the bound FMN, the concentration of pure FMN and FAD was increased from 10 µM to 90 µM, using a linear gradient, for 10 min. The injection volume and total run time were 20 µL and 5 min, respectively. The detection wavelength was set to 267 nm. The retention times of FMN and FAD were 4.2 ± 0.1 min and 3.6 ± 0.1 min, respectively.

### Assays of chromate reductase and nitroreductase activity of NfoR

The activity of chromate reductase was measured using a photometric assay, based on the detection of residual Cr (VI) in the reaction, as described previously^4^. Meanwhile, we also determined the effects of Cu (II) and Fe (III) on NfoR activity. Briefly, the reaction mixtures (1 mL each), supplemented with 50 mM Tris-HCl buffer (pH 7.0), 20 µM NfoR, 0.3 mM NADH, 0.2 mM Cr (VI), and 50 µM or 100 µM Cu (II) and Fe (III), were incubated for 5 min at 37 °C. A control sample without NfoR was assayed to rule out the reaction between Cr (VI) and NADH. Reaction mixtures, with Cu (II) concentrations ranging from 0 to 600 µM, were tested to evaluate the effect of Cu (II) on the stability of NfoR. The roles of other divalent metals, such as Cd, Co, Mn, Ni, Pb, and Zn, in the enzyme activity, were also tested. A reaction mixture, without divalent metals, was used as control.

For the nitroreductase activity assay, ammonium nitrate and nitrobenzene were used as substrates. The 500 μL reaction mixtures [50 mM Tris-HCl buffer [pH 7.0], 10 μM NfoR, 0.2 mM NADH, 100 μM ammonium nitrate or nitrobenzene, and different concentrations of Cu (II)] were incubated for 5 min at 28 °C. The residual oxidized NADH concentration was measured at 345 nm, at 5 s intervals, using a UV-VIS spectrophotometer.

### Spectral measurements of NfoR

Fluorescence measurements were conducted in 2.0 mL Tris-HCl buffer (pH 7.0) containing NfoR and its mutants (5.0 × 10^−6^ mol L^−1^), and Cu (II) concentrations of 0, 10, 20, 30, 40, 50, 70, or 100 × 10^−6^ mol L^−1^, incubated at 37 °C for 5 min. Besides, we detect the binding between NfoR with Fe(III) or Ni(II). The analysis of fluorescence spectra was performed using a LS55 spectrofluorometer (Perkin Elmer). Fluorescence emission spectra, ranging from 300 to 450 nm, were detected using the slit width of 10 nm excitation and emission silt and the excitation wavelength of 280 nm.

For the measurements of UV-Vis absorption spectra, 2-mL reaction mixtures, containing 20 mM Tris-HCl buffer (pH 7.0), 5 μM NfoR, and different concentrations of Cu (II), were incubated for 30 min at 37 °C. UV/Vis spectra, ranging from 190 to 400 nm, were determined at 25 °C.

The Far-UV CD spectra of NfoR and its variants were recorded using a Jasco J-85 spectropolarimeter, equipped with software-driven Peltier-based temperature controller. The cell path was set at 0.1 cm.

### Isothermal titration calorimetry (ITC)

ITC measurements were performed at 25 °C using a MicroCal iTC200 (GE Healthcare). Proteins and ligands were buffered with 20 mM Tris-HCl (pH 7.0) and thoroughly degassed before use. Titrations were performed using 0.1 mM NfoR with 10 mM Cu (II), Ni (II) or Fe (III). Each ITC run comprised an initial injection of 0.4 μL followed by 19 × 2 μL injections of NfoR into the sample cell. The experiments were performed in triplicate. Data were analyzed with Origin 7.0 software by fitting a titration curve to the corrected data using a single-site interaction model.

### Site-directed mutagenesis and purification of NfoR

Site-directed mutagenesis was determined using the Quick-change Lightning Site Directed Mutagenesis Kit (Takara) according to the manufacturer’s protocol. The plasmid was digested using *Nco*I and *Xho*I restriction enzymes (Takara) and sub-cloned into a fresh pET-28a vector. The resulting plasmid was verified using DNA sequencing. The single point mutants (H100G, H128G, C163S and M165G), double mutants (H100GH128G, H100GM165G and H128GM165G), and triple mutants (H100GH128G M165G) were prepared using the above-mentioned method. The expression and purification of mutants have been described previously in the section of His-tagged NfoR purification.

### Enzyme model of recombinant NfoR

The X-ray crystal structure of a putative NAD(P)H-flavin oxidoreductase (PDB ID:2HAY) from *Streptomyces* was downloaded from the PDB website (http://www.pdb.org/pdb/home/home.do), which showed the highest identities with NfoR (45%). Because detailed and mechanical knowledge about NfoR is lacking, the structure of *Streptomyce*s NAD(P)H-flavin oxidoreductase was used as the template for modeling. Neighbor-joining trees, for publically available NfoR homologues across all bacterial species, were generated using Mega (v.8.1). All the protein structures were mapped by Pymol (http://www.pymol.org/).

### Statistical analyses

All of the data are presented as mean ± s.d. When more than two groups were compared, one-way analysis of variance (ANOVA), followed by Duncan’s comparison of means, was used. *P* < 0.05 was considered statistically significant.

### Data availability

The authors declare that the data, supporting the findings of this study, are available within the manuscript and its Supplementary Information files, or from the corresponding author on request.

## Electronic supplementary material

Supplementary Information
