# Supplementary material for: Copper (II) binding of NAD(P)H- flavin oxidoreductase (NfoR) enhances its Cr (VI)-reducing ability
Source: Sci Rep. 2017 Nov 13;7:15481. doi: 10.1038/s41598-017-15588-y (PMC5684319; doi:10.1038/s41598-017-15588-y)
Supplement: Supplementary file 1 — Supplementary Information [file 41598_2017_15588_MOESM1_ESM.doc]

Copper (II) binding of NAD(P)H- flavin oxidoreductase (NfoR) enhances its Cr (VI)-reducing ability

Huawen Han^a,1^, Zhenmin Linga^a,1^, Tuoyu Zhou^1^, Rong Xu^1^, Yongxing He^1^, Pu Liu^2^, Xiangkai Li^1^*

^1^Ministry of Education, Key Laboratory of Cell Activities and Stress Adaptations, School of Life Science, Lanzhou University, Tianshui South Road #222, Lanzhou, Gansu 730000, People’s Republic of China

^2^Department of Development Biology Sciences, School of Life Science, Lanzhou University, Tianshui South Road #222, Lanzhou, Gansu 730000, People’s Republic of China

* Corresponding author:

Phone: +86-931-8912560

Fax: +86-931-8912561

E-mail address: [xkli@lzu.edu.cn](mailto:xkli@lzu.edu.cn)

^a^These authors contributed equally to this work.

**Table S1 Strains and plasmids used in this study**

| **Strains or**  **plasmids** | **Relevant genotype** | **Reference**  **or Source** |
| --- | --- | --- |
| Strains |  |  |
| LZ-01 | Wild-type | Isolation |
| RN4220 | 8325-4 r- initial recipient for modification of plasmids which are introduced into *S.aureus* from *E. coli* | Refer[^1^](#_ENREF_1) |
| Δ*nfoR* | LZ-01 *nfoR::kan* | This study |
| CnfoR | LZ-01 *nfoR::kan* pLInfoR | This study |
| DH5α | Clone host strain, *supE44*ΔlacU169  (*φ80dlacZΔM15*) *hsdR17 recA1*  *endA1gyrA96 thi-1 relA1* | This study |
| Rosseta(DE3) | F-ompThsdSB(rB^-^mB^-^) gal dcm (DE3)pRARE2, Cm^r^ | This study |
| Plsmids |  |  |
| pMD-18T | Clone vector, Amp^r^ | Takara |
| pET-28a(+) | Expression vector with a hexahistidine tag, Kan^r^ | Novagen |
| pE*nfoR* | pET28a(+) with the *nfoR* coding sequence, Kan^r^ | This study |
| PE*nfoR*ΔH100G | pET28a(+) with the *nfoR* mutants, Kan^r^ | This study |
| PE*nfoR*ΔH128G | pET28a(+) with the *nfoR* mutants, Kan^r^ | This study |
| PE*nfoR*ΔC163S | pET28a(+) with the *nfoR* mutants, Kan^r^ | This study |
| PE*nfoR*ΔM165G | pET28a(+) with the *nfoR* mutants, Kan^r^ | This study |
| PE*nfoR*ΔH100GΔH128G | pET28a(+) with the *nfoR* mutants, Kan^r^ | This study |
| PE*nfoR*ΔH100GΔM165G | pET28a(+) with the *nfoR* mutants, Kan^r^ | This study |
| PE*nfoR*ΔH128GΔM165G | pET28a(+) with the *nfoR* mutants, Kan^r^ | This study |
| pMAD | Shuttle vector, temperature sensitive, Ap^r^ Em^r^ | Refer[^1^](#_ENREF_1) |
| pMAD*nfoR* | pMAD containing upstream and downstream fragments of *nfoR* and *kan* gene, for *nfoR* mutagenesis, Ap^r^ Kan^r^ Em^r^ | This study |
| pL150 | Shuttle vector, Ap^r^Cm^r^ | Refer[^2^](#_ENREF_2) |
| pL*nfoR* | pLI50 with *nfoR* ORF and its promoter,Ap^r^ Cm^r^ | This study |

Note: Kan^r^, kanamycin-resistant; Ap^r^, ampicillin-resistant; Cm^r^, chloramphenicol-resistant; Em^r^, erythromycin-resistant.

**Table S2 Relevant primers used in this study**

| **Primer** | **Sequence (5’→3’)** |  |
| --- | --- | --- |
| *nfoR*-F | CCCATGGGCCATCATCATCATCATCACATGAGCAATATGAATCAAACAATTAT | This study |
| *nfoR*-R | CCGCTCGAGTTCTTTTGGTCCAACCCATT | This study |
| Up-*nfoR*-F | CCGGGAGCTCGAATTCAAATCAGGTGTATTGATGACTGC | This study |
| Up-*nfoR*-R | CAAAATCCCTTAACGTGAGTTGAACATAGCCTCCAATTTTTATAT | This study |
| nptII-F | ACTCACGTTAAGGGAT | This study |
| nptII-R | CAGGTGGCACTTT TCGGGGA | This study |
| Down-*nfoR*-F | TCCCCGAAAAGTGCCACCTGATAGAATACAGTATGTCTAAATATATAAAATTAAA | This study |
| Down-*nfoR*-R | GGGCGATATCGGATCCTATCAATTCATTTTCTTTTCTGGTA | This study |
| pMAD-F | TCAGACGGTTCGATCTTGCTC | This study |
| pMAD-R | CTGGACAGCATGGCCTGCA | This study |
| *nfoR* H100G F | CGTATGTACAAGGCATGTTAAGAGA | This study |
| *nfoR* H100G R | GTGATCTTGACGTTACATTTTTACG | This study |
| *nfoR* H128G F | GCAGATTTCGGTATTTCTGATAATG | This study |
| *nfoR* H128G R | TTGGAATGCATCGAATTTTTGT | This study |
| *nfoR* M165G F | ATTGAAGGTTTTAGTCTGGATACAGTG | This study |
| *nfoR* M1658G R | CGGACATGAATCAATACCTAACAAT | This study |
| *nfoR* C163S F | TCTCCGATGGAAGGTTTTAGTCT | This study |
| *nfoR* C163S R | TGAATCAATACCTAACAATGCGG | This study |

**Table S3 Dissociation constants and related thermodynamic parameters of NfoR for Cu(II) and Ni(II)**

| **Protein** | **Metal** | **N** | ***Kd*(µM)** | **Δ*H*(kJ/mol)** | **Δ*S* (J/mol/K)** |
| --- | --- | --- | --- | --- | --- |
| NfoR | Cu(II) | 5.26±0.09 | 85.5 | -25.73±0.64 | -67.7 |
| NfoR | Ni(II) | ND | ND | ND | ND |

ND: No detectable interaction.

**
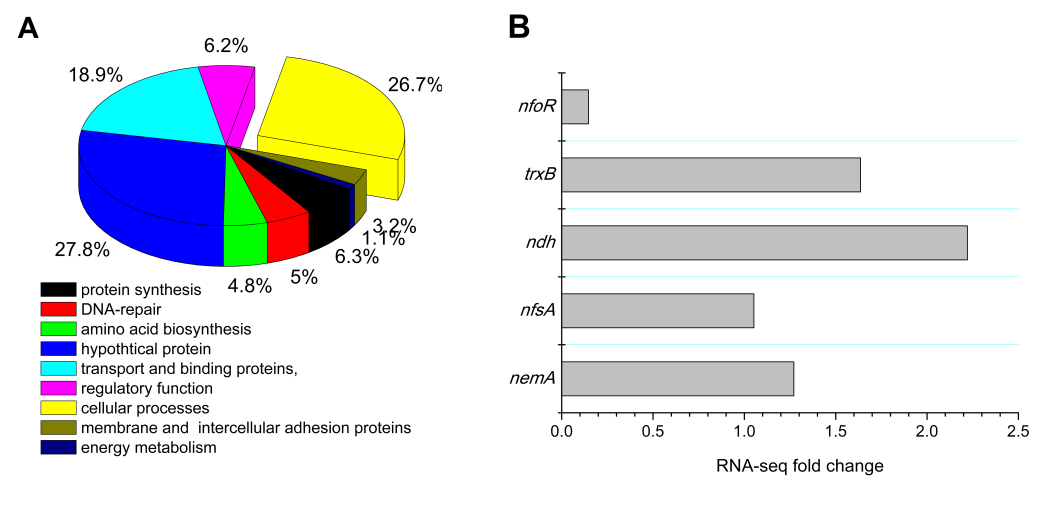
**

**Fig.S1 A. The upregulated genes of *S.aureus* LZ-01 under 400****µM Cr (VI) stress. B. Putative upregulated gene annotated as encoding chromate reductase based on transcriptome.**

**
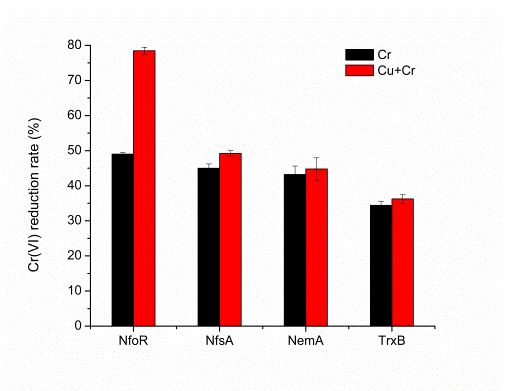
**

**Fig. S2 The enzyme activities of different chromate reductase using Cr (VI) as substrates in the absence or presence of Cu(II) .**


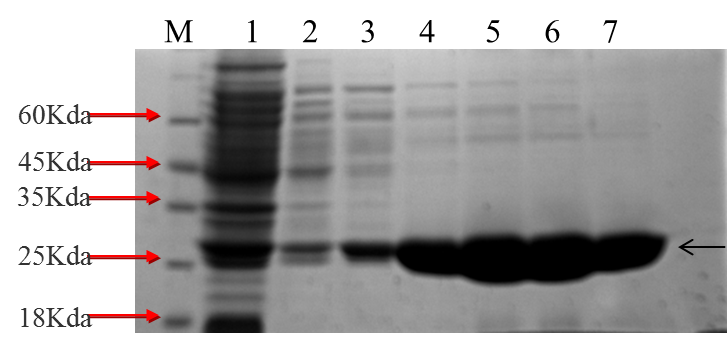


**Fig.S3 Affinity purification of NfoR with a N-terminal 6×His tag. Proteins were resolved on 12% SDS-PAGE gels and stained with Coomassie blue.** **The sizes of molecular mass markers are indicated in kDa on the left.**

**
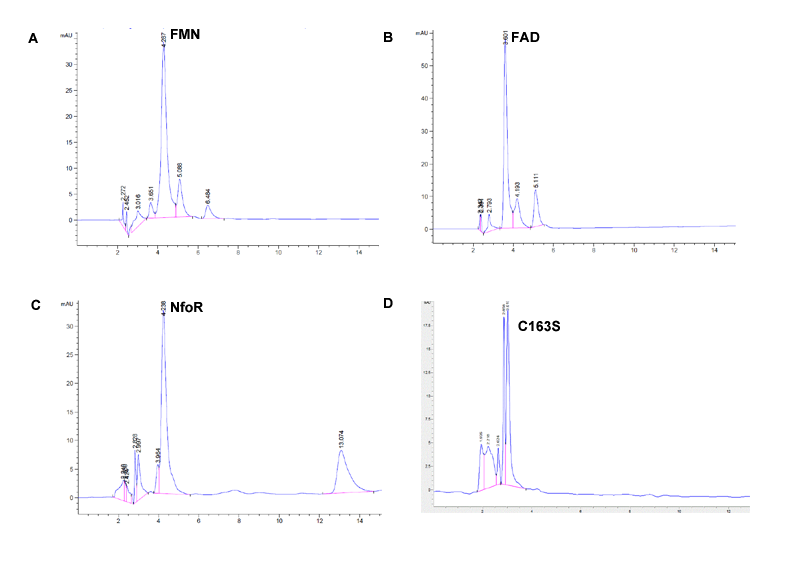
**

**Fig.S4 Analysis of flavin composition of FAD (A), FMN (B), NfoR supplement (C) and C163S supplement (D) by HPLC**

**
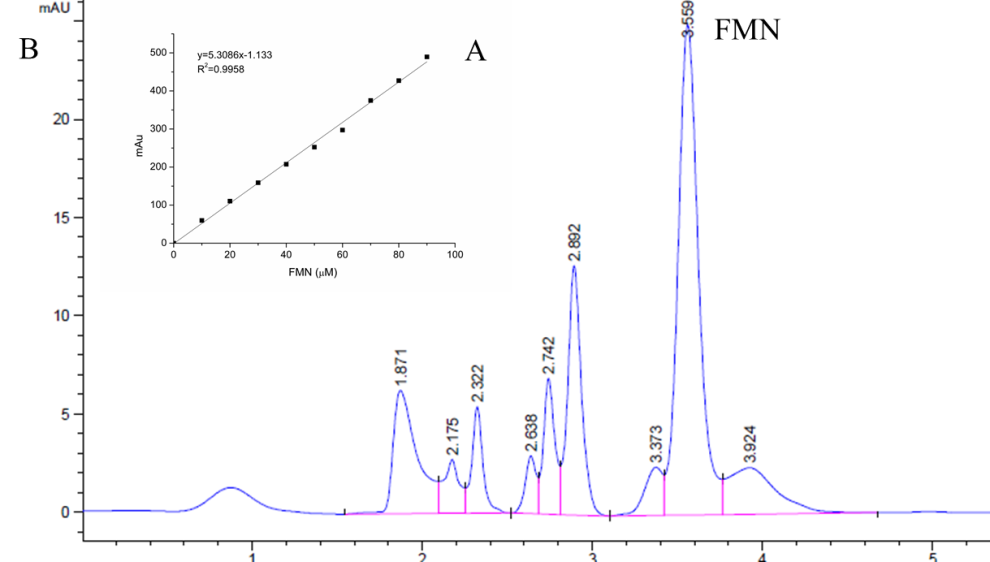
**

**Fig.S5 A, The correlation between the FMN concentrations and the mAu. B. the FMN content of isolated NfoR.**

**
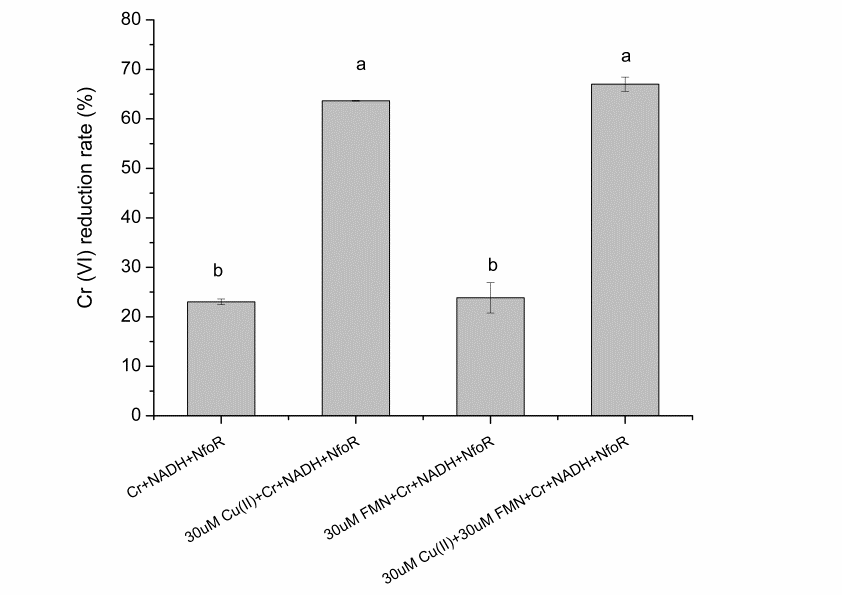
**

**Fig.S6 Supplementation free FMN has no significant effect on NfoR activity.**

**
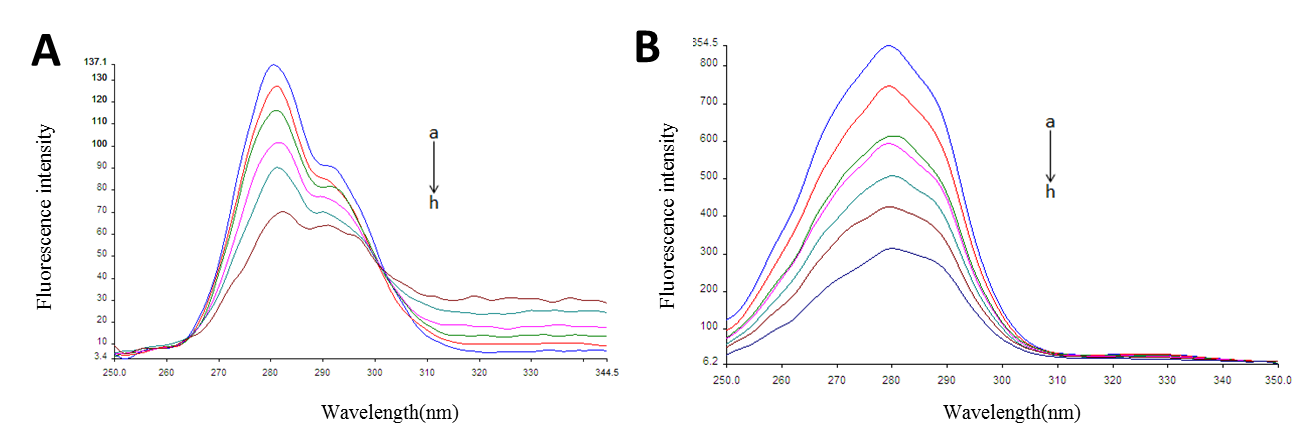
**

**Fig.S7 Synchronous fluorescence spectrum (Δλ = 15 nm (A) and Δλ = 60 nm (B)) (a) 5×10^–6^ mol L^–1^ NfoR; (b)-(h) 5×10^–6^ mol L^–1^ NfoR in the presence of 0, 10, 20, 30,40, 50, 70 and 100×10^–6^ mol L^–1^ Cu(II).**

**
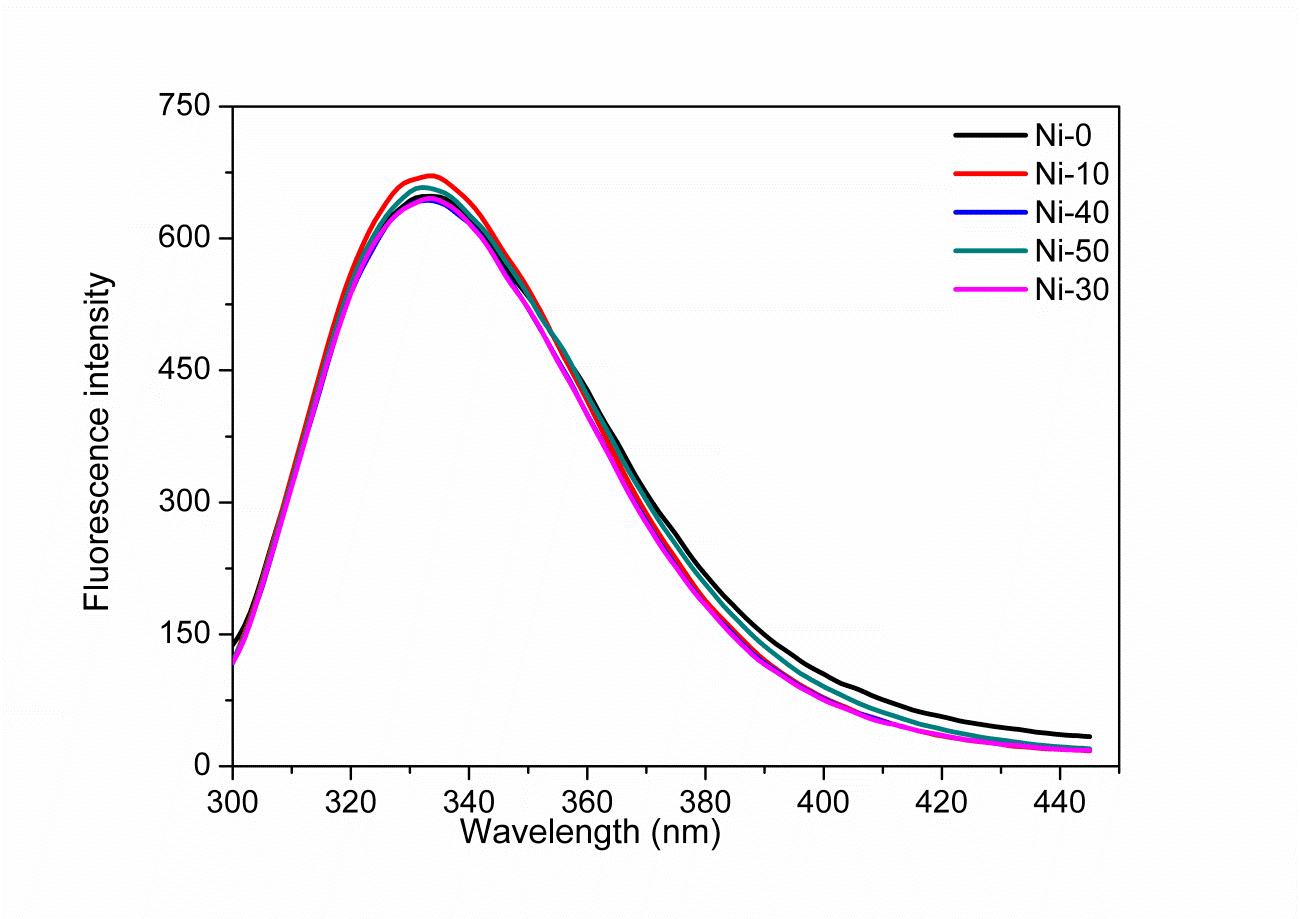
**

**Fig.S8 Fluorescence emission spectra of NfoR excited at 280 nm in the presence of Ni (II), NfoR concentration (5×10^–6^ mol L^–1^), Ni (II) concentration (0, 10, 30, 40 and 50 ×10^–6^ mol L^–1^).**

**
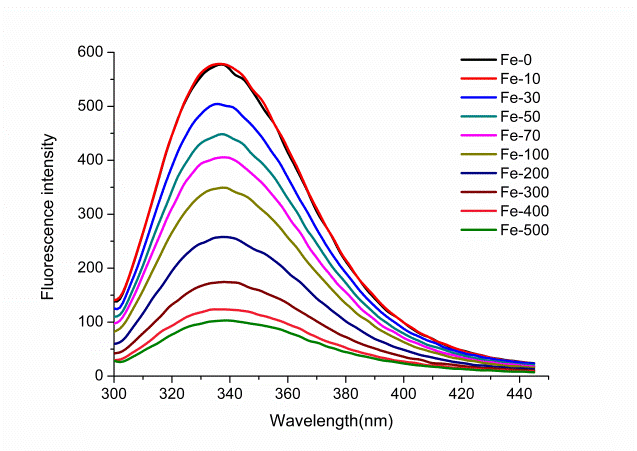
** **
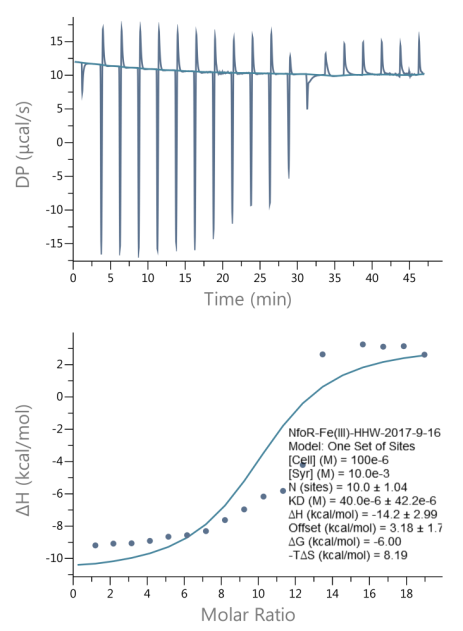
**

**Fig.S9 A, Fluorescence emission spectra of NfoR excited at 280 nm in the presence of Fe(III) NfoR concentration (5×10^–6^ mol L^–1^), Fe(III) concentration (0, 10, 30, 50, 70, 100, 200,300, 400 and 50 0 ×10^–6^ mol L^–1^ ). B. Isothermal calorimetry (ITC) analyses of NfoR. Approximately 10 mM Fe (III) was injected into 0.1 mM NfoR.**

**
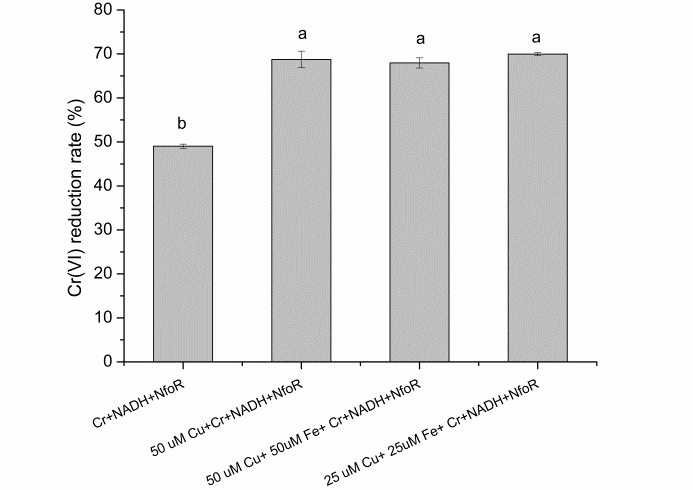
**

**Fig.S10 The competition influence of Fe (III) and Cu (II) on NfoR activity. Reaction mixtures (1 mL) containing 20 mM Tris-HCl buffer (pH 7.0), 20 μM NfoR, 0.3 mM NADH, 0.2 mM Cr(VI), and 25μM and 50μM Cu(II) or Fe (III), were incubated for 8 min in 37℃. Error bars indicate standard errors.**


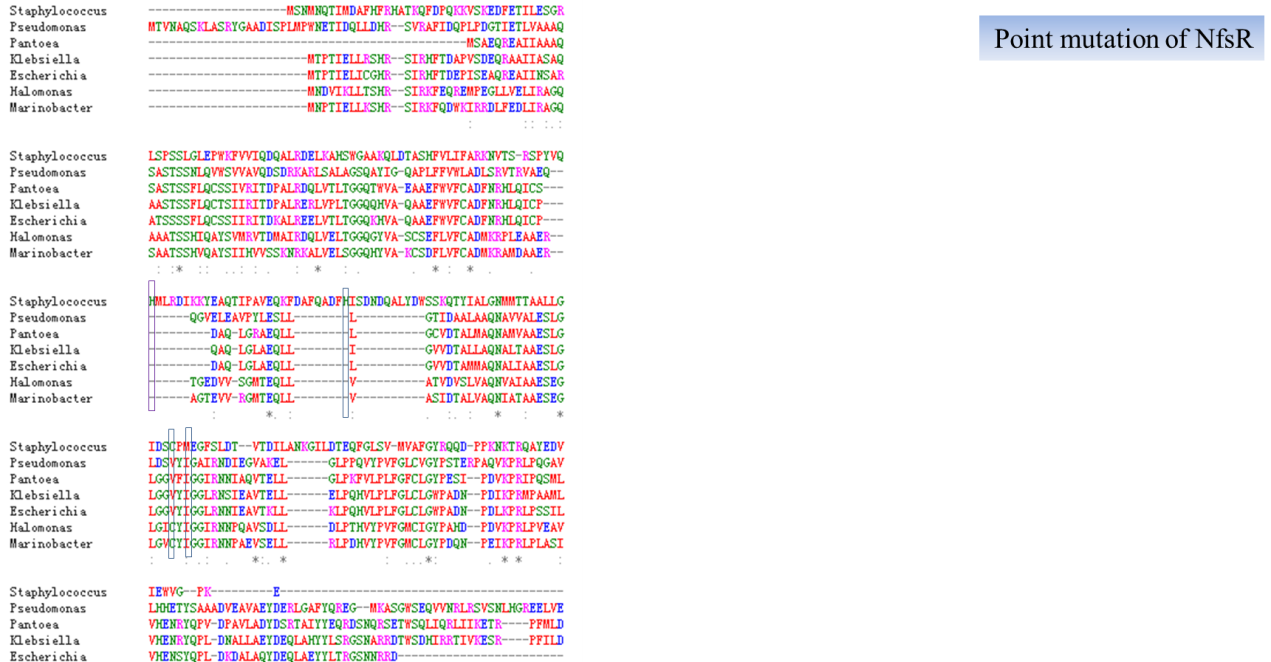


**Fig.S11 Sequence alignments of NfoR homologues. Asterisks indicate identical residues; colons, residues with a high level of similarity; and a period, residues with lower similarity. Rectangle the represents the targeted residues were chosen as mutation sites.**

**
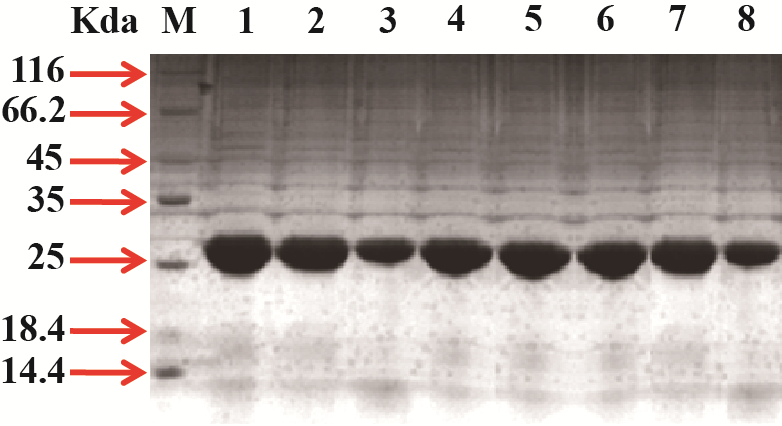
**

**Fig.S12 SDS-PAGE analysis for NfoR mutants. Lane1-8 represented H100G, H128G, C163S, M165G, H100GH128G, H100G M165G, H128G M165G, H100GH128GM165G, respectively The sizes of molecular mass markers are indicated in kDa on the left.**

**
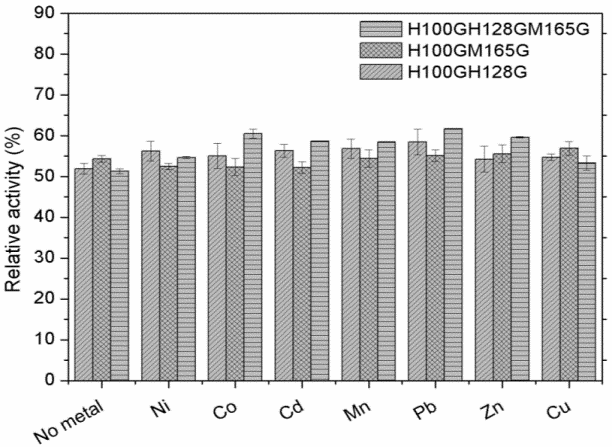
**

**Fig.S13 The effects of different metals on the activity of NfoR mutants.**

1 Baker, J., Sengupta, M., Jayaswal, R. K. & Morrissey, J. A. The Staphylococcus aureus CsoR regulates both chromosomal and plasmid-encoded copper resistance mechanisms. *Environmental microbiology* **13**, 2495-2507, doi:10.1111/j.1462-2920.2011.02522.x (2011).

2 Haipeng Sun, Y. Y., Ting Xue and Baolin Sun. Modulation of cell wall synthesis and susceptibility to vancomycin by the two-component system AirSR in Staphylococcus aureus NCTC8325. *BMC Microbiology* **13**, 10, doi: 10.1186/1471-2180-13-286 (2013).
